# Comparison of Gene Editing versus a Neutrophil Elastase Inhibitor as Potential Therapies for *ELANE* Neutropenia

**DOI:** 10.33696/immunology.4.129

**Published:** 2022

**Authors:** Vahagn Makaryan, Merideth Kelley, Breanna Fletcher, Isabella Archibald, Tanoya Poulsen, David Dale

**Affiliations:** University of Washington, Department of Medicine, Seattle, Washington, USA

**Keywords:** CRISPR/Cas9, G-CSF, Myeloid, *ELANE* KO, Neutrophil elastase, Inhibitors, Neutropenia

## Abstract

Heterozygous mutations in *ELANE*, the gene for neutrophil elastase, cause cyclic and congenital neutropenia through the programed cell death of neutrophil progenitors in the bone marrow. Granulocyte colony-stimulating factor is an effective therapy for these diseases, but alternative therapies are needed, especially for patients who do not respond well or are at high risk of developing myeloid malignancies. We developed an HL60 cell model for *ELANE* neutropenia and previously demonstrated that transient and regulated expression of mutant *ELANE* causes cell death by accelerated apoptosis. Knocking down the mutant gene or exposure to a potent inhibitor of neutrophil elastase rescued neutrophil development. Because of the great diversity in causative *ELANE* mutations, we generated stable HL60 clones expressing mutant P139L, C151Y and G214R and compared the effects of elastase inhibitor exposure to an *ELANE* knock-out line on cell development and function. ATRA induced differentiation demonstrated comparably impaired myeloid cell development for all three lines with upregulated expression of GRP78/BIP, an abnormality corrected by exposure of these cells to the elastase inhibitor MK-0339. The inhibitor and KO of mutant *ELANE* led to formation of neutrophils with comparable chemotactic and bactericidal capacities. We concluded that both strategies have great potential for the treatment of cyclic and congenital neutropenia. However, an orally absorbed, cell permeable inhibitor of neutrophil elastase, if proven safe and effective in a clinical trial, might be the better alternative to G-CSF or gene editing to treat *ELANE* neutropenia.

## Introduction

Mutations in *ELANE*, the gene for neutrophil elastase (NE), are the most common cause of cyclic and severe congenital neutropenia. These are very serious diseases, predisposing patients to severe bacterial infections. Both conditions are treated effectively now with recombinant human granulocyte colony-stimulating factor (G-CSF), but this treatment requires daily or alternate day subcutaneous injections and carries the potential risk of stimulating development of myeloid leukemia. Currently hematopoietic stem cell transplantation is the only other effective treatment; thus, there is a need for alternative therapies [1–11].

HL60 cells are an established model for study of myeloid cell development. We have used this model to demonstrate that transient and regulated expression of mutant *ELANE* in HL60 cells interrupts myeloid differentiation and causes cell death via accelerated apoptosis in cells of the myeloid lineage. We have also demonstrated that an inhibitor of enzymatic activity of neutrophil elastase (NE), developed by Merck-DuPont pharmaceuticals in the 1980s, partially corrected the defect induced by mutant *ELANE* expression in these cells [12]. In collaboration with Nasri et al., we recently reported that knockdown of *ELANE* in HL60 cells expressing the mutant gene can restore myeloid cell development and functions [13]. These results suggest that both NE specific inhibitors and genetic manipulation (*ELANE* KO or mutation correction) are promising novel treatments for *ELANE* associated neutropenia. Because of the importance of these results for patients and because of the diversity of mutations in *ELANE* that cause these diseases, we have expanded our studies in HL60 lines expressing different *ELANE* mutants and comparing the effects of the elastase inhibitor versus knockout (KO) of *ELANE* on myeloid cell differentiation and function.

We analyzed the therapeutic effects of MK-0339 NE inhibitor and genetic *ELANE* KO by examining two key characteristics of myeloid cells that underline in the basis of normal hematopoiesis: cell survival and cell differentiation. To evaluate the safety, i.e., retention of critical functions, with these two therapeutic approaches (NE inhibitor and genetic KO) we examined the antimicrobial capacity of these cells by measuring chemotaxis and bacterial killing.

## Materials and Methods

HL60 cells (kindly provided by Dr. Steve Collins, Fred Hutchinson Cancer Research Center, Seattle, WA). Cell lines were maintained at 37°C 5% CO_2_ incubator in RPMI supplemented with 10% fetal bovine serum (Avantor Seradigm), 2 mM L-glutamine and Pen Strep (Thermo Fisher Scientific). Myeloid differentiation of HL60 cells was stimulated by adding 2 uM all-trans retinoic acid (ATRA) and culturing for 6 days. NE inhibition was achieved by addition of 1 uM MK-0339 in the culture on the first day of differentiation [12].

### Cytospins and staining

8.0×10^4^ HL60 cells differentiated as above were spun onto Cytoslide microscope slides (ThermoFisher) using a Cytospin 4 low speed cytocentrifuge (Thermo Scientific), then stained with the Kwik-Diff staining system (Thermo Shandon) following manufacturer’s recommendations. Microphotographs were taken on a LEITZ LABORLUX S polarizing light microscope at 400X magnification using a Nikon DSLR digital camera.

### Generation of ELANE mutant P139L, C151Y, G214R and *ELANE* KO HL60 cell lines

We used CRISPR/Cas9 gene editing technology with homology directed repair (HDR) to generate mutant *ELANE* knock-in (KI) P139L, C151Y and G214R HL60 stable cell clones. We used Zhang lab’s CRISPR Designtool (http://crispr.mit.edu/) to identify a guide RNA to target near the desired mutation with minimal off-target effects. This guide was cloned into the pSpCas9(BB)-2A-GFP vector (Addgene 48138).

The Guide-it sgRNA Screening Kit (Clontech) was used to test the efficacy of different single guide RNAs (sgRNAs) *in vitro* prior to using them in HL60 cells. A 100nt single-stranded DNA oligonucleotide (ssODN) was designed as an HDR repair template, containing the desired mutation with ∼50 nt of homology on either side of the predicted cut site. HL60’s were co-transfected with the ssODN repair template and the Cas9 vector containing the sgRNA using electroporation (Neon transfection system, ThermoFisher). Cas9 expression was confirmed by fluorescence microscopy using anti-Cas9 polyclonal and biotinylated Goat anti-Rabbit secondary antibodies (Epigentek) with AlexaFluor 488 streptavidin detection. 24 hours after transfection, the cells were single-cell sorted using GFP selection into two 96 well plates containing 50% conditioned media. The cells were allowed to expand for three weeks. After the first week, the media was refreshed every 2–3 days. After three weeks of growth, a direct PCR kit (Clontech) was used to screen the colonies and confirm mutations.

*ELANE* total KO was performed by using a custom designed human gene knock-out kit for *ELANE* (GKO_HS3_*ELANE*) produced by Synthego. Clonal selection and identification were performed as described above for *ELANE* KI clones.

### Flow cytometry analysis

The survival rate of cells during myeloid differentiation was evaluated by labeling with APC conjugated annexin V and PI (Biolegend) and analyzed by FACS. Cell debris and necrotic cells were gated out, and the percentage of annexin V-positive cells was expressed as the number of apoptotic cells/total number of cells.

Myeloid differentiation of HL60 cells was analyzed by flow cytometry using CD11b-APC (130-110-554) and CD15-VioBlue (130-113-488) cell surface antibodies (Miltenyi Biotec). Cell debris was gated out by using a zombie yellow viability kit (Biolegend, 423103)

### Western blot analysis

NE protein loss in *ELANE* KO clones was confirmed by western blot analysis using anti-NE monoclonal antibody (Abcam, #121260, secondary: Abcam #205718) with actin specific monoclonal antibody as a loading control (Santa Cruz, #47778, secondary: VWR #NA931V). The unfolded protein response (UPR) master regulator protein GRP78/BiP was detected by western blot analysis using anti-BiP rabbit antibody (Cell Signaling, 3183S, secondary #7074).

### Chemotaxis assay

6.5 mm Transwell plates with 5.0 µm pore inserts (Corning, #3421) were used to evaluate chemotaxis. IL-8 at 10 ng/ml was added to the lower chamber as the chemoattractant. 3 ×10^4^ HL60 cells differentiated for 6 days were placed in the upper chamber. Cells that had migrated through the membrane were counted in the lower chamber after 20 min of incubation.

### Bacterial killing assay

A real-time luminescence based bacterial killing assay was developed to compare killing ability by differentiated granulocytes. HL60 cells were differentiated for 6 days as described above. The resultant granulocytes were incubated in a white 96 well flat-bottom tissue culture plate (Costar, #3917) at 1×10^5^ or 5×10^4^ cells per well in the presence of 2×10^5^ luxCDABE-expressing bacterial cells. *E. coli* strain DH5alpha transformed with a modified bacterial luciferase gene (luxCDABE) from Photorhabdus luminescens30 (addgene, #14080) was opsonized in HBSS CaCl2^+^ MgCl2^+^, 20% FBS for 30 minutes at 37^o^C with slow rotation in a hybridization oven (Fisher Scientific, #13-247-20). The opsonized bacteria were diluted to an OD of 0.04 in HBSS CaCl2^+^, MgCl2^+^ 10% FBS and added to differentiated HL60’s at a 2:1 ratio. The plate was returned to the hybridization oven and incubated for 2.5 hours. Light emission in relative light units (RLUs), was measured using a luminometer (CentroXS3 LB960). Wells without differentiated HL60s (*E. coli* only) and with 10 ng/ml of the phagocytosis inhibitor Cytochalasin D (Santa Cruz Biotec, #sc-201442) served as controls. Changes in RLU were calculated and graphed.

## Results

**Figure 1:**
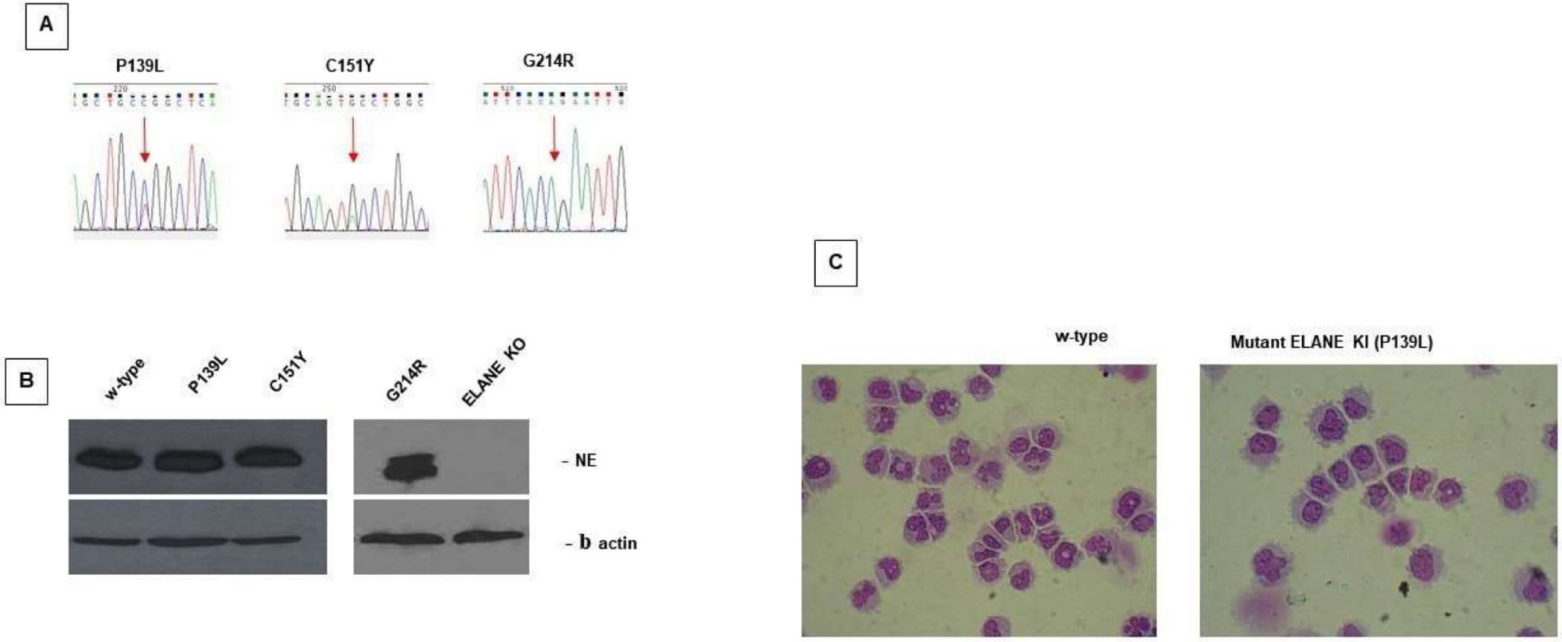
*ELANE* knock-in (KI) and Knock-out (KO) HL60 cell lines. **A.** Electropherograms depicting *ELANE* P139L, C151Y and G214R mutations in HL60 lines. **B.** Human promyelocytic HL60 cell lines expressing WT, mutant NE and no NE (KO) were induced to differentiate with 2 μM ATRA (all-trans retinoic acid), maintained 5 d in culture, lysed and Western blots were performed using anti-NE and anti-actin Abs as described in Materials and methods. **C.** HL60 cell lines expressing either WT or P139L mutant NE cultured and differentiated as described above. Cell cytospins stained with Kwik-Diff were imaged using a Nikon digital camera. Cell differentiation was evaluated by light microscope.

We first generated mutant *ELANE* KI P139L, C151Y and G214R HL60 stable cell clones. The heterozygous amino acid substitutions in P139L and C151Y are consistent with naturally occurring mutations in patients with *ELANE* associated neutropenia. The mutant G214R cell line harbors a homozygous substitution, which was intentionally used in this study to see the cumulative effect of the mutant protein produced by both alleles in the complete absence of the normal NE (Figure 1A). Interestingly, the expression of the NE in these mutant cell lines is very similar to the w-type. Using a commercially available customizable gene knock-out (KO) kit (Synthego), we also successfully developed an HL60 cell line completely lacking w-type NE for these comparative studies (Figure 1B).

The resultant cell lines, in the undifferentiated state, demonstrate very similar cell growth and cell viability in culture compared to the w-type. ATRA induced myeloid differentiation of these mutant cell lines during 6 days in culture results in a typical block of differentiation in the promyelocytic stage and accelerated apoptosis which correlates with what is observed in patients with *ELANE* associated neutropenia (Figure 1C).

### Cell survival

**Figure 2:**
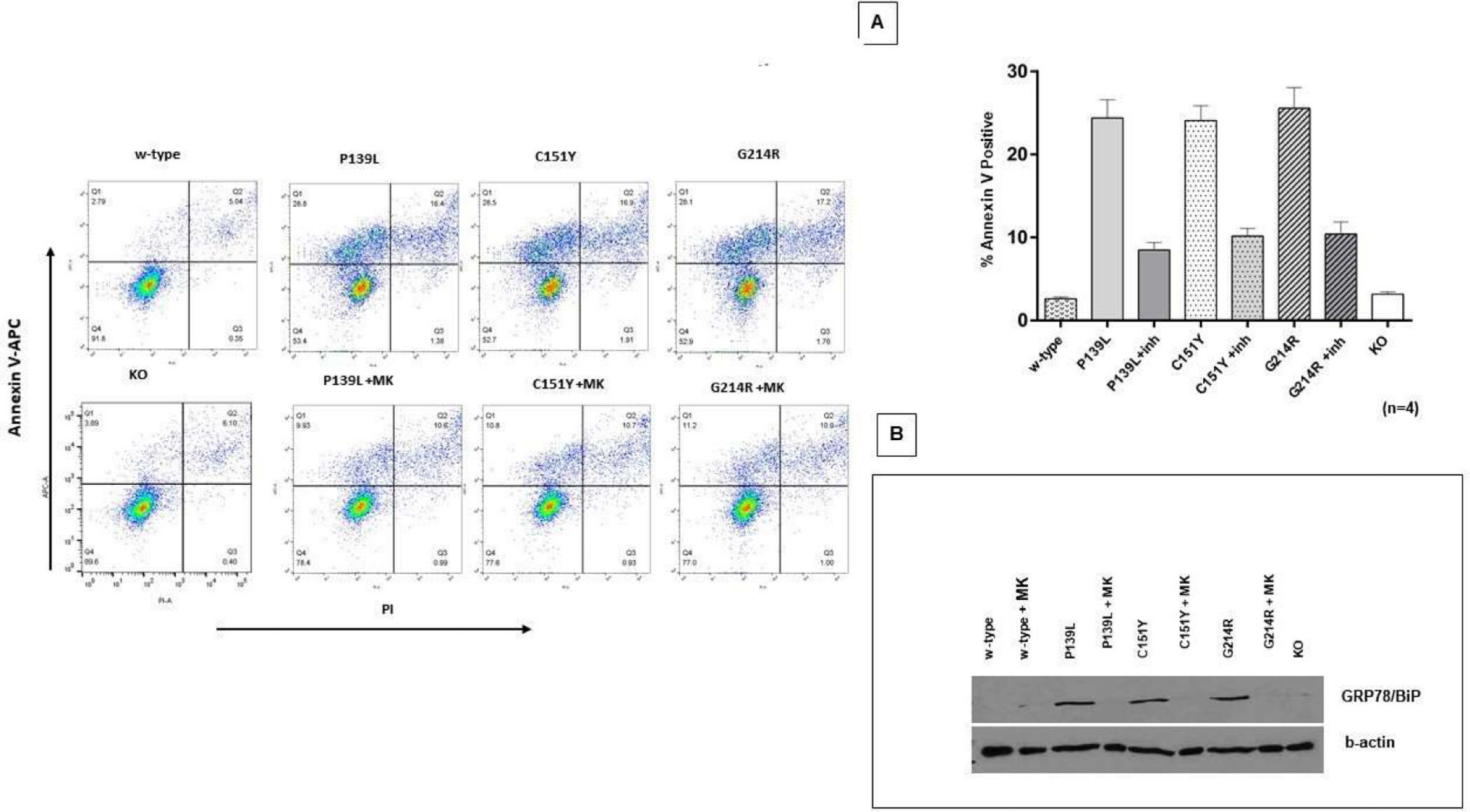
Effect of MK-0339 inhibitor and *ELANE* KO on cell survival. **A.** Cells cultured for 5 days in the presence or absence of 1uM MK-0339 inhibitor were labeled with annexin V and analyzed using flow cytometry. The proportion of early apoptotic (Q1) annexin V–positive cells is indicated. Representative experiment histograms are shown. *N* =4. **B.** Cells cultured as described above were lysed and western blots probed with anti-BiP and anti-actin Abs as described in materials and methods.

CRISPR/Cas9 edited *ELANE* KI and KO lines, as well as w-type HL60 cells, were cultured with ATRA as described above to induce myeloid differentiation. At day 5, cells were labeled with Annexin V and cell apoptosis was measured by flow cytometry. As expected, accelerated apoptosis was observed in all three *ELANE* mutant cell lines (P<0.0001) compared to the w-type. Addition of 1 uM MK-0339 significantly reduced the impaired cell survival. (P<0.0001). Survival of the *ELANE* KO line was very similar to w-type (Figure 2A). The unfolded protein response (UPR) in these cell lines was assessed by western blotting using a polyclonal antibody to GRP78/BiP. GRP78/BiP protein expression was noticeably upregulated in all three mutant cell lines compared to w-type. Addition of 1uM MK-0339 normalized this abnormality in these cell lines. GRP78/BiP protein expression in the KO cell line was not altered compared to w-type (Figure 2B).

### Differentiation

**Figure 3:**
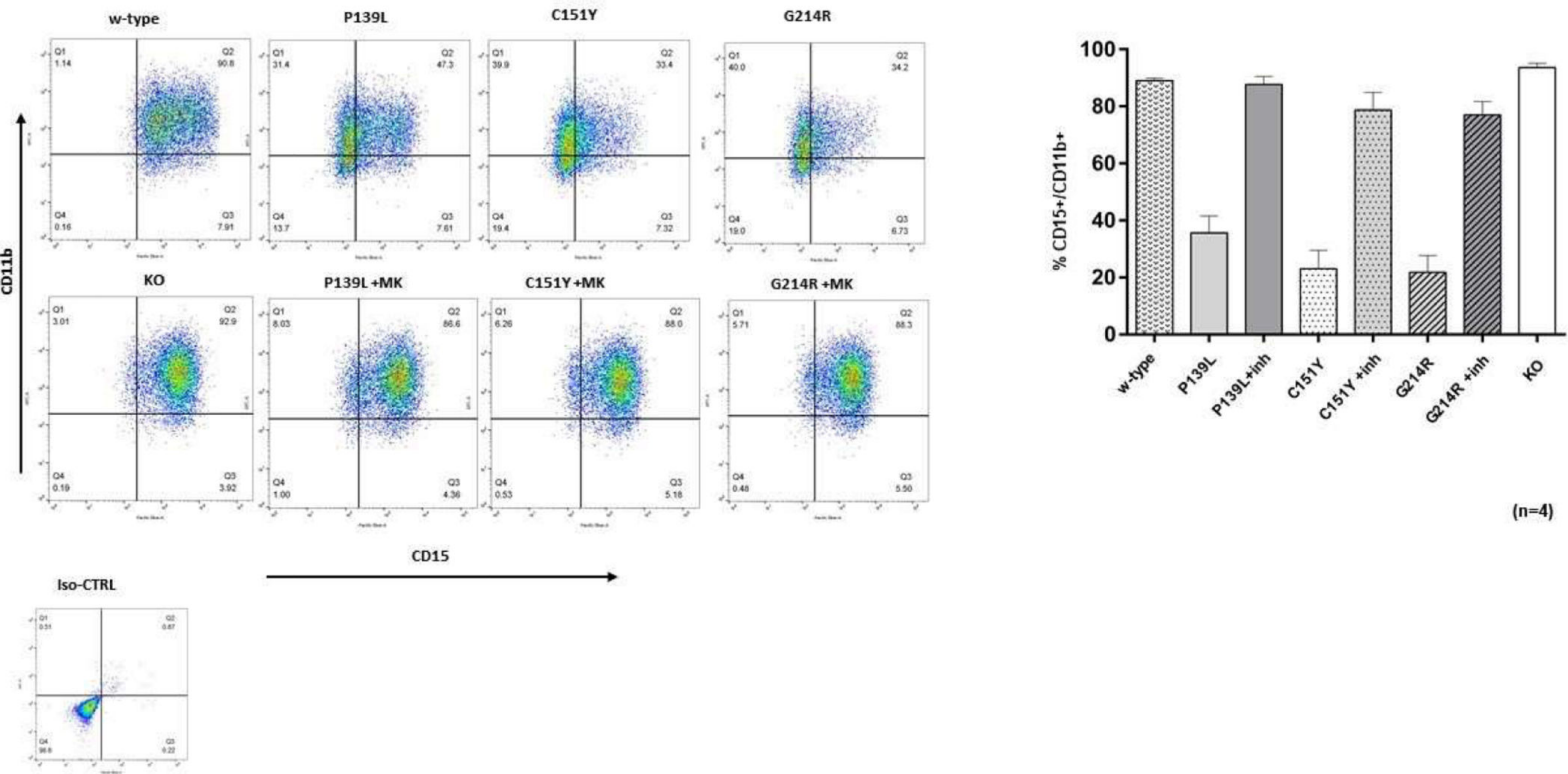
Effect of MK-0339 inhibitor and *ELANE* KO on cell differentiation. Cells cultured for 5 days in the presence or absence of 1 uM MK-0339 inhibitor were labeled with antibodies to CD11b and CD15 surface markers and analyzed using flow cytometry. The proportion of CD11b^+^/CD15^+^ positive cells (Q2) is indicated. Representative experiment histograms are shown. *N* = 4.

CRISPR/Cas9 edited *ELANE* KI and KO lines, as well as the w-type CTRL HL 60 cells were treated with 2 uM ATRA to induce myeloid differentiation and cultured as described above. At day 5, cells were labeled with CD11b and CD15 granulocytic differentiation surface markers and examined by flow cytometry. The proportion of CD11b^+^/CD15^+^ cells was significantly lower in all three mutant cell lines compared to the w-type (P<0.0001), showing impairment of myeloid differentiation consistent with that observed in patients with *ELANE* associated neutropenia. Addition of 1 uM MK-0339 restored the impaired cell differentiation. There were no significant differences in the proportions of cells expressing these surface markers between the w-type and *ELANE* KO cell lines (Figure 3).

### Chemotaxis

**Figure 4:**
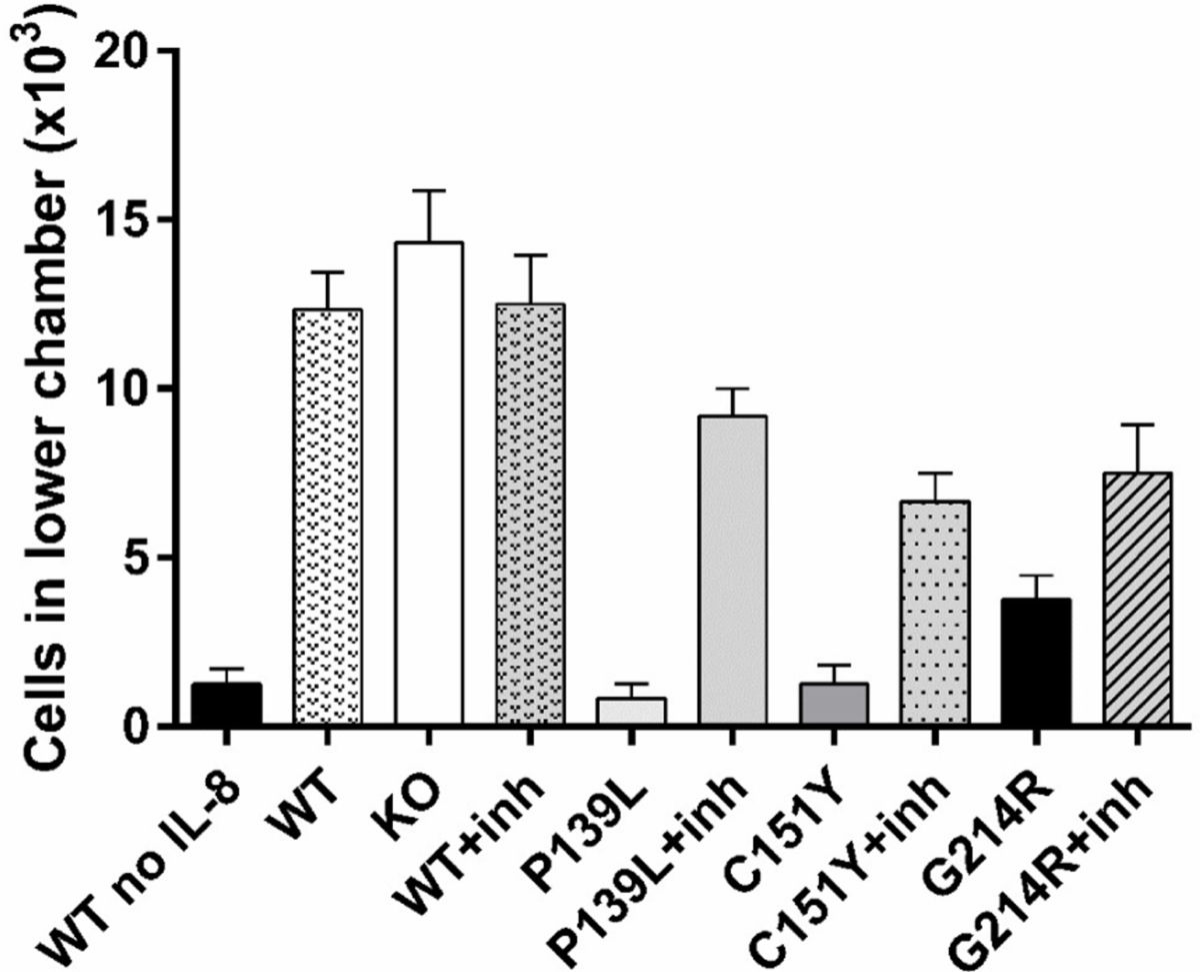
Effect of MK-0339 inhibitor and *ELANE* KO on cell chemotaxis. CRISPR/Cas9 edited *ELANE* KI cell lines cultured with or without 1 uM MK-0339 and KO lines, as well as the w-type CTRL HL60 cells were cultured for 6 days in the presence of 2 uM ATRA to induce myeloid differentiation. Motility of the resultant cells was assessed toward 10 ng/ml IL-8 using a transwell system. *N* = 3.

CRISPR/Cas9 edited *ELANE* KI lines cultured with or without MK-0339, the KO line, and w-type CTRL HL60 cells were cultured for 6 days in the presence of 2uM ATRA to induce myeloid differentiation. The migration of the resultant cells toward 10 ng/ml IL-8 was examined using a transwell system. Chemotaxis was significantly diminished in all three mutant lines (p<0.0002), and was partially restored by treatment with MK-0339. There were no statistically significant alterations in chemotactic capacities of *ELANE* genetic KO or MK-0339 inhibitor treated HL60 cells compared to untreated w-type (P>0.0831) (Figure 4).

### Bacterial killing assay

**Figure 5:**
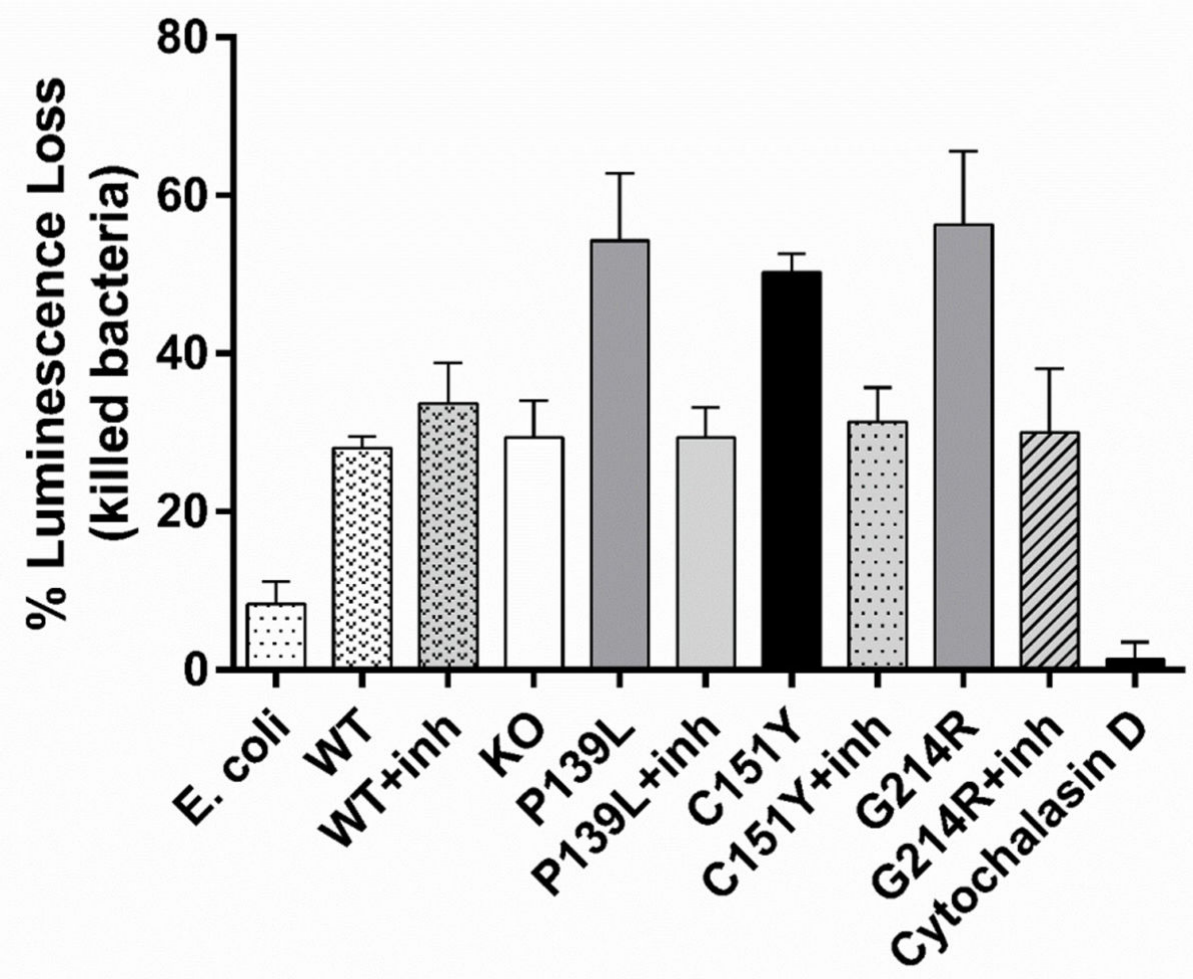
Effect of MK-0339 inhibitor and *ELANE* KO on bacterial killing. CRISPR/Cas9 edited *ELANE* KI cell lines cultured with or without 1uM MK-0339 and KO lines, as well as the w-type CTRL HL60 cells were cultured for 6 days in the presence of 2 uM ATRA to induce myeloid differentiation. Bacterial killing capacity was measured by luciferase reporter assay using the luxCDABE gene cluster modified *E. coli* bacterial strain and a luminometer. *N* = 3.

CRISPR/Cas9 edited *ELANE* KI lines cultured with or without MK-0339, the KO line, and w-type CTRL HL60 cells were cultured for 6 days in the presence of 2uM ATRA to induce myeloid differentiation. The bacterial killing capacity of these cells was examined using the luxCDABE gene cluster modified *E. coli* strain as a luciferase reporter. We observed no significant differences in the bactericidal capacities of inhibitor treated and KO cell lines compared to the w-type (P>0.9996) (Figure 5). Inhibitor-free mutant lines exhibited higher killing than inhibitor treated lines, KO and w-type, but this difference was only statistically significant for the G214R line compared to w-type (p=0.03) (See Discussion).

## Discussion

Human promyelocytic HL60 cells are an established model for studying myeloid cell development and diseases. HL60 cells grow well in basic tissue culture medium with a cell doubling time of two days. They demonstrate high efficiency and recovery rates during transfection (electroporation). In the presence of DMSO or all trans retinoic acid (ATRA) HL60 cells easily differentiate towards functional granulocytes in 4–6 days, with antimicrobial characteristics and morphology comparable to human neutrophils [14–20].

Bone marrow cells from patients with cyclic and severe congenital neutropenia, particularly from patients with mutations associated with leukemia or poor response to G-CSF, are not readily available. The diseases are rare, bone marrow aspiration is painful and expensive, and patients with mutations causing more severe disease, e.g., G214R and C151Y, are usually transplanted or die from infections or leukemia. Mouse models of *ELANE* associated neutropenia have failed to recapitulate the human phenotype or serve as a model for the disease. There is an increasing interest in using the zebrafish platform as a model for this disorder [21,22]. We have previously successfully used patient derived iPS cells for studying neutropenia [12]. Although these cells are directly derived from patients, there is always a concern about the artificial nature of the methods used to produce them. Tissue culture techniques with these cell lines are very complex and results can vary significantly. Lineage differentiation assays using iPSCs are lengthy and do not always consistently result in homogeneous cell production [23–27]. Regardless of their availability and ease of use, HL60 cells have limitations when studying human hematopoiesis. HL60 is a leukemic cell line with acquired proliferative characteristics due to mutations in some key tumor suppressor genes. Undifferentiated HL60 cells lack secondary granules and are missing important granule and surface marker proteins specific to mature neutrophils. With differentiation in DMSO or ATRA, these cells acquire antimicrobial capacity, but these functional properties are significantly less than for normal blood neutrophils [18–20,28]. In this study we have observed another potential limitation of working with HL60 cells. When examining the bacterial killing capacity of *ELANE* mutant KI lines differentiated using ATRA we detected higher bacterial killing in cultures without added inhibitor (Figure 5). We hypothesize that this is because culture with ATRA alone induces partial differentiation followed by cell death in the mutant-expressing lines. The dying cells release enzymes and cellular DNA in a “NET effect” may contribute to the bacterial killing. It is also possible that apoptotic cells have more adhesive cell membranes, causing the bacteria to stick to the cell surface which reduces the luminescence signal.

CRISPR/Cas9 gene editing techniques have made precise manipulation of the genome feasible. The current study required creation of stable cell lines harboring mutant *ELANEs* or *ELANE* KO clones. This would not have been possible using primary bone marrow cells. Using single cell sorting, we have developed stable HL60 cell lines expressing P139L, C151Y and G214R single point *ELANE* mutations. Although it has been reported that the total amount of NE protein is lower in cells of neutropenic patients [29], our *ELANE* KI mutant cell lines express an equal amount of NE compared to w-type. This enables these lines to express sufficient mutant protein to result in measurable phenotypic changes. These specific amino acid substitutions were chosen based on clinical studies of *ELANE* associated neutropenia. The P139L mutation usually manifests a mild phenotype compared to C151Y and G214R, which are known to cause severe disease with frequent complications and a high probability of transformation to MDS/AML. [6] It is important to note that the G214R expressing cell line harbored a homozygous mutation. Naturally occurring homozygous *ELANE* mutations have not been observed in the human population. Interestingly, we did not observe a more severe phenotype in this cell line compared to the other mutant lines. This could be due to the confining conditions of *in vitro* experiments vs the natural bone marrow environment in patents, or the limiting conditions of the HL60 cellular platform.

It is important to note that when cultured in the immature state, the CRISPR/Cas9 edited mutant *ELANE* HL60 clones used in this study demonstrate very similar characteristics in terms of cell growth and cell viability compared to w-type. ATRA induced myeloid differentiation over 6 days promotes transition to a specific neutropenic phenotype in these mutant cell lines which correlates with what is observed in patients with *ELANE* associated neutropenia.

We have previously reported that the *ELANE* KO strategy improves survival of HL60 cells expressing mutant NE. We have also observed that NE inhibitors have similar effects [12]. Nasri et al., recently reported that CRISPR/Cas9 mediated *ELANE* KO improves myeloid differentiation and maturation in neutropenia patient derived bone marrow primary cells [13].

Another approach for genetic correction of *ELANE* associated neutropenia is the CRISPR/Cas9 mediated direct correction of *ELANE* mutations utilizing the cell’s homology directed repair (HDR) machinery. At first glance, this methodology is very promising as it repairs the nucleotide mutation and does not result in *ELANE* KO. However, there are some serious limitations to this approach. First, the efficiency of HDR after CRISPR/Cas9 mediated double-strand break is very low and might not be feasible for clinical applications. Secondly, *ELANE* heterozygous gain-of-function mutations are distributed throughout all five exons of the gene, and it would require specific guide and repair templates for each patient-specific genotype. There is also a serious concern with widely used adeno-associated viral (AAV) delivery methods of the donor repair template and its use for clinical applications. Moreover, a gene editing approach focused on the correction of *ELANE* mutations may result in novel mutations due to low efficiency and inadequate homology dependent repair of double strand cuts and thus to novel variants of NE protein.

In this study we compared *ELANE* KO cells expressing no NE to cells expressing mutant NE in the presence of the cell permeable NE inhibitor, MK-0339. In general, the phenotype rescue effects were similar. The inhibitor improved cell survival and proliferation for cells expressing any of the 3 mutations. The inhibitor was also permissive of maturation for all 3 cell lines. The effect was very similar to the KO cell line. There was no significant difference in survival or myeloid differentiation between the *ELANE* KO line compared to w-type. These results suggest that either knocking out both alleles of the *ELANE* gene or inhibiting NE with a cell permeable inhibitor might have similar clinical benefits. We acknowledge that we have examined only 3 of the more than 100 different *ELANE* mutations associated with cyclic and congenital neutropenia. We believe they represent the diversity of mutations, but will continue to investigate other mutations and the basic mechanisms underlying the biologic effects of neutrophil elastase inhibitors.

The two knock-down approaches used in these experiments result in either total removal of NE (genetic KO) or greatly diminished enzymatic function (chemical inhibitor). Therefore, we assessed whether granulocyte effector functions were impaired in differentiated KO or inhibitor treated mutant NE lines. Chemotaxis and bactericidal assay results with KO and inhibitor treated lines were similar to w-type. It may be surprising that KO and inhibition of neutrophil elastase have no effect on bactericidal activity. It is obvious that the current study and previous studies have examined only a few organisms and biological processes which involve neutrophils and their proteases. It is also unclear whether NE or other of the related proteases have effects on remodeling of myeloid precursors as they progress from early progenitors to mature neutrophils.

With the total KO strategy, there is a valid concern for the complete elimination of normal NE. *ELANE* associated neutropenia is an autosomal dominant disease and there is a functional normal NE product translated from the healthy allele. Total *ELANE* KO completely eliminates the expression of both healthy and mutant variants of NE protein. There are a number of contradictory studies in the literature about the role of NE. Some investigators have demonstrated a non-redundant role of NE protein, showing it has an essential role in antimicrobial defense during innate immunity by degrading outer bacterial membrane proteins and modulating inflammatory cytokines [30–33]. NE knock-out mice displayed increased susceptibility to sepsis due to infection with gram negative bacteria [34]. NE also has a role in antifungal defense [35]. There is data that NE takes an important role during the formation of neutrophil extracellular traps (NETs). It has been demonstrated that *ELANE* KO, and more interestingly, NE inhibitor treated granulocytes, exhibit abnormal NET formation [36]. Other investigators state that the antimicrobial role of NE is highly redundant and there are a number of other serine proteases expressed by white blood cells that have similar functions during innate immune response [37–39]. The highlight is Papillon-Lefevre Syndrome (PLS) which is the only known human disorder causing NE deficiency. This is a rare autosomal recessive genetic disease caused by mutations in the *DPPI* gene encoding cysteine protease cathepsin C/dipeptidyl peptidase I (DPPI). This enzyme mediates N-terminal processing of NE as well as other serine proteases: cathepsin G and Proteinase 3. PLS patients display a severe reduction in the activity and stability of all three serine proteases. Interestingly, patients with PLS have no bacteriocidic deficiencies suggesting the redundant role of these serine proteases for killing common bacteria [40].

The other serious concern for the *ELANE* KO strategy is off target effects. Even with recent improvements it has been demonstrated that CRISPR/Cas9 genome editing can hit off target sites, generate DNA double strand breaks and introduce unintended mutations [41]. The use of computational bioinformatics along with next generation sequencing (deep sequencing) is essential for reducing off target effects and selecting suitable editing components in order to minimize the occurrence of off target effects.

Considering the disputed role of NE in host immune defense mechanisms, as well as the comparable results of NE inhibition vs *ELANE* KO observed in this study, we believe that pharmacological inhibition of NE is more favorable and more readily feasible for clinical applications than a genetic KO approach. By its nature, chemical inhibition is to some degree incomplete and in many cases is reversible. With chemical inhibition, the manipulation is on the protein level but not on the genetic level. The gene remains functional and there should always be some level of normal NE product expressed, which would be well controllable by administered dose correction of the NE inhibitor.

There is a long history of studying NE inhibitors as anti-inflammatory drugs. We first reported the potential use of these inhibitors to treat *ELANE* associated neutropenia more than a decade ago. Steadily we and others have accumulated increasing evidence that cell permeable inhibitors may be an effective treatment, but thus far there have been no clinical trials to demonstrate clinical effects or benefits. The dosing strategies to achieve clinically beneficial inhibitory concentrations in the bone marrow are not known. The *in vitro* concentrations of the inhibitor used in this study (MK0339), however, are comparable to the blood concentrations achieved in previous clinical trials in normal subjects and patients with various inflammatory diseases [42–44]. Thus, we believe there is sufficient evidence from our *in vitro* work and previous clinical trials to justify small scale, short-term phase 2 investigations of this inhibitor and possibly other NE inhibitors.

It is still very early in the development of alternatives to G-CSF as treatments for *ELANE* associated neutropenia. Work advancing the knockout strategy is advancing rapidly. These studies involve either knocking out both alleles to produce KO cells similar to those used in this investigation or single allele knock out to remove only the mutant allele [45,46]. Clinical applications will require leukapheresis for collection of patients’ hematopoietic stem cells, *ex vivo* editing and autologous transplantation with sufficient numbers of progenitor cells to produce sufficient neutrophils to correct neutropenia. There is precedent for being successful with each of these steps toward permanent correction of neutropenia. Successful application will also depend upon avoidance of significant off target effects of the gene editing procedures. In the near term, it would be best to conduct at least a preliminary trial of an orally absorbed, cell permeable inhibitor of neutrophil elastase.

## Conclusion

We developed an HL60 cell model to study the mechanisms of and potential treatments for *ELANE* neutropenia. We studied three stable HL60 clones expressing *ELANE* mutants, i.e., P139L, C151Y and G214R, mutations associated with different clinical outcomes. All three mutant clones produced few mature neutrophils on exposure to ATRA, with similar apoptosis profiles. These abnormalities were abrogated by adding the cell permeable elastase inhibitor MK-0339 to the cultures. Both treatment of the mutant clones with this inhibitor and *ELANE* KO led to formation of neutrophils with comparable chemotactic and bactericidal capacities. These studies suggest that both strategies have great potential for the treatment of cyclic and congenital neutropenia. We conclude that an orally absorbed, cell permeable inhibitor of neutrophil elastase, if proven safe and effective in a clinical trial, is a good alternative to G-CSF or gene editing to treat *ELANE* neutropenia.
